# Deep learning-based multifeature integration robustly predicts central lymph node metastasis in papillary thyroid cancer

**DOI:** 10.1186/s12885-023-10598-8

**Published:** 2023-02-08

**Authors:** Zhongzhi Wang, Limeng Qu, Qitong Chen, Yong Zhou, Hongtao Duan, Baifeng Li, Yao Weng, Juan Su, Wenjun Yi

**Affiliations:** 1 Department of General Surgery, the Affiliated Zhuzhou Hospital Xiangya Medical College, Central South University, Zhuzhou, Hunan China; 2 Department of General Surgery, The Second Xiangya Hospital of Central South University, No. 139, Renmin Central Road, Changsha, 410011 P.R. China; 3 Department of Ultrasound Diagnosis, the Affiliated Zhuzhou Hospital Xiangya Medical College, Central South University, Zhuzhou, Hunan China; 4 Department of Metabolic Endocrinology, the Affiliated Zhuzhou Hospital Xiangya Medical College, Central South University, Zhuzhou, Hunan China; 5 Department of Medical Administration, the Affiliated Zhuzhou Hospital Xiangya Medical College, Central South University, No.116, Changjiang South Road, Zhuzhou, 412007 P.R. China

**Keywords:** Papillary thyroid cancer(PTC), Central lymph node metastasis(CLNM), *BRAF V600E* gene mutation, Convolutional neural network(CNN)

## Abstract

**Background:**

Few highly accurate tests can diagnose central lymph node metastasis (CLNM) of papillary thyroid cancer (PTC). Genetic sequencing of tumor tissue has allowed the targeting of certain genetic variants for personalized cancer therapy development.

**Methods:**

This study included 488 patients diagnosed with PTC by ultrasound-guided fine-needle aspiration biopsy, collected clinicopathological data, analyzed the correlation between CLNM and clinicopathological features using univariate analysis and binary logistic regression, and constructed prediction models.

**Results:**

Binary logistic regression analysis showed that age, maximum diameter of thyroid nodules, capsular invasion, and *BRAF V600E* gene mutation were independent risk factors for CLNM, and statistically significant indicators were included to construct a nomogram prediction model, which had an area under the curve (AUC) of 0.778. A convolutional neural network (CNN) prediction model built with an artificial intelligence (AI) deep learning algorithm achieved AUCs of 0.89 in the training set and 0.78 in the test set, which indicated a high prediction efficacy for CLNM. In addition, the prediction models were validated in the subclinical metastasis and clinical metastasis groups with high sensitivity and specificity, suggesting the broad applicability of the models. Furthermore, CNN prediction models were constructed for patients with nodule diameters less than 1 cm. The AUCs in the training set and test set were 0.87 and 0.76, respectively, indicating high prediction efficacy.

**Conclusions:**

The deep learning-based multifeature integration prediction model provides a reference for the clinical diagnosis and treatment of PTC.

**Supplementary Information:**

The online version contains supplementary material available at 10.1186/s12885-023-10598-8.

## Significance statement

This study used a fine needle puncture for sampling and prediction simultaneously during the preoperative examination, and innovatively established prediction models based on genetic mutations and clinicopathological factors that can be used to evaluate CLNM of PTC with high sensitivity and specificity, thus achieving the ideal combination of molecular and traditional diagnostic methods for clinical application. The high diagnostic efficacy of this model can help clinicians to diagnose metastases in the central lymph nodes of PTC, thus avoiding prophylactic central neck dissection in patients with negative lymph nodes and reducing the incidence of complications such as laryngeal nerve injury and hypoparathyroidism. These prediction models facilitate the formulation of individualized patient treatment plans and assisting clinicians in accurately diagnosing the disease.

## Introduction

Papillary thyroid carcinoma (PTC) is the most common thyroid malignancy, accounting for approximately 84% of all thyroid cancers [1, 2]. Cervical lymph node metastasis in PTC is closely related to the local recurrence of tumors, disease-free survival (DFS), and overall survival (OS). The mode and scope of surgery are determined by the presence of lymph node metastasis and the site of metastasis. Central lymph node metastasis (CLNM) is the primary cervical lymph node metastasis site in PTC [3–5].

Few tests can diagnose CLNM of PTC with high accuracy. Preoperative ultrasound has high diagnostic value in detecting lateral neck lymph node metastasis (LLNM) but is less sensitive in diagnosing CLNM [6, 7]. The accuracy of intraoperative palpation for CLNM by surgeons is usually less than 30% [8]. However, in PTCs with lymph nodes larger than 1 cm in diameter, the rate of CLNM is as high as 40%-90%. Even in microscopic thyroid cancer, the rate of CLNM can be 25%-45% [9–11]. Prophylactic central neck dissection (PCND) reduces the risk of recurrence but increases the incidence of complications such as recurrent laryngeal nerve injury and hypoparathyroidism [12, 13]. Therefore, PCND and the extent of dissection in patients with PTC are controversial [14–16]. There is an urgent need for a marker that can be used to accurately predict CLNM or establish a predictive model for CLNM.

Genetic sequencing of tumor tissues helps improve the understanding of tumor genomes and enables the development of personalized cancer treatments for patients with certain genetic variants [17]. *BRAF V600E* is the most commonly mutated gene in PTC [18]. The *BRAF V600E* mutation is a useful reference for predicting the presence of CLNM and could be used to determine whether to perform PCND in patients with clinically lymph node-negative (cN0) stage PTC [19–21]. However, the predictive value of *BRAF V600E* mutation in CLNM needs to be confirmed by further studies (with large patient cohorts) based on the clinicopathological characteristics of and geographical differences in CLNM.

In this study, we included 488 patients diagnosed with PTC by ultrasound-guided fine-needle aspiration biopsy, collected clinicopathological data, analyzed the correlation between CLNM and clinicopathological features using univariate analysis and binary logistic regression, and constructed a nomogram prediction model and a convolutional neural network (CNN) prediction model of CLNM. The model was also validated in the subclinical metastasis group and the clinical metastasis group. This study provides a new method for predicting CLNM in patients with PTC.

## Methods

### Patient selection

**Fig. 1:**
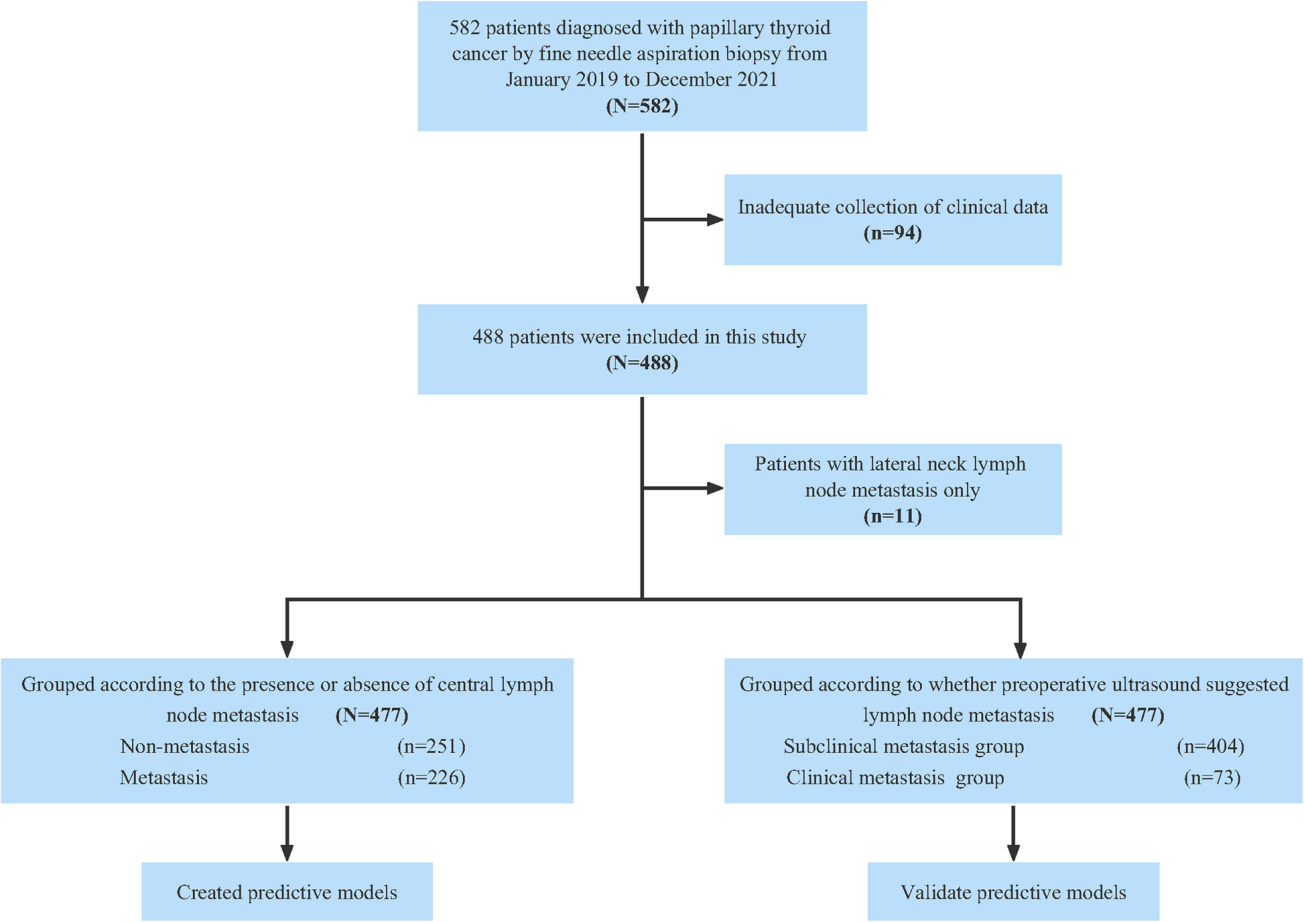
Flowchart of patient selection for the study

From January 2019 to December 2021, a total of 582 patients suspected of having PTC after ultrasound-guided fine-needle aspiration biopsy of thyroid nodules were recruited for this study. The inclusion criteria were as follows: (a) preoperative ultrasonography of the thyroid gland and cervical lymph nodes, ultrasound-guided fine-needle aspiration cytology of thyroid nodules, and fine-needle aspiration tissue DNA (FNAT-DNA) *BRAF V600E* gene mutation testing, with the aspiration biopsy considered to be PTC; (b) first thyroid surgery, with the surgical approach following the "Thyroid Cancer Diagnosis and Treatment Standard" issued by the Medical Administration of the Chinese Health Care Commission; (c) complete genetic information, ultrasound findings and clinicopathological data of the patients; and (d) intraoperative rapid pathology and postoperative pathology confirming PTC. The exclusion criteria were as follows: (a) a history of thyroid-related medication; (b) preoperative treatment with neck radiation and iodine-131; and (c) recurrence of the tumor and presence of distant metastases. Among the patients, 94 patients with incomplete clinical data were excluded, and 488 patients were finally included in this study. The median age was 45 years, 128 patients were male, and 360 patients were female (Fig. 1, Table 1).

**Table 1:** Basic and comprehensive characteristics in this study

Characteristics	Overall N(%)
Sample size	488
**Sex**	
Male	128 (26.2)
Female	360 (73.8)
**Age (years)**	
< 45	240 (49.2)
≥ 45	248 (50.8)
**Combined benign thyroid disease**	
No	262 (53.7)
Nodular goiter	138 (28.3)
Lymphocytic thyroiditis	67 (13.7)
Both	21 (4.3)
**Surgical method**	
Unilateral	213 (43.6)
Total	275 (56.4)
***BRAF V600E* gene mutation**	
No	99 (20.3)
Yes	389 (79.7)
**Lymph node metastasis**	
No	251 (51.4)
Central	173 (35.5)
Lateral	11 (2.2)
Both	53 (10.9)
**Maximum diameter of thyroid nodules**	
< 10 mm	210 (43.0)
10–20 mm	204 (41.8)
> 20 mm	74 (15.2)
**Nodule location**	
Upper 1/3	102 (20.9)
Middle 1/3	146 (29.9)
Lower 1/3	77 (15.8)
Isthmus	63 (12.9)
Whole	100 (20.5)
**Aspect Ratio**	
< 1	284 (58.2)
≥ 1	204 (41.8)
**Microcalcification**	
No	127 (26.0)
Yes	361 (74.0)
**Nodule boundary**	
Clear	199 (40.8)
Blurred	289 (59.2)
**Capsular invasion**	
No	275 (56.4)
Close	65 (13.3)
Invasion	148 (30.3)
**Number of lesions**	
Unifocal	346 (70.9)
Multifocal	142 (29.1)
**ACR-TIRADS classification**	
3	20 (4.1)
4a	274 (56.1)
4b	153 (31.4)
4c	27 (5.5)
5	12 (2.5)
6	2 (0.4)

All patients were operated on by the same team of thyroid surgeons. All patients underwent thyroid lobectomy and ipsilateral CLND on the side where the nodule was located or total thyroidectomy and bilateral CLND if the lesion was present on both sides; some patients underwent lateral lymph node dissection(LLND) based on preoperative evaluation, intraoperative rapid frozen section reports, and intraoperative conditions. Of the 488 patients included in the study, 213 patients underwent unilateral thyroidectomy, 275 patients underwent total thyroidectomy, and 73 patients underwent LLND. All pathological specimens were sent to the pathology department for paraffin fixation and histological analysis. All specimens were examined microscopically and cross-checked by two or more experienced pathologists for analysis and lymph node metastasis evaluation, and a final diagnostic report was given. According to the postoperative pathological findings, 251 patients did not have lymph node metastasis, 173 patients had CLNM, 11 patients had LLNM, and 53 patients had both CLNM and LLNM (Table 1).

### Clinicopathological features

Clinicopathological data, such as sex, age (45 years [median age] was used as the grouping criterion), combined benign thyroid disease, surgical method, *BRAF V600E* gene mutation results, cervical lymph node metastasis and ultrasound characteristics (maximum diameter of thyroid nodule, nodule location, nodule aspect ratio, nodule microcalcification, nodule boundary, capsular invasion, number of lesions and ACR-TIRADS classification), were collected from all included patients (Table 1).

### Detection of *BRAF V600E* gene mutation status by second-generation sequencing

The specimens extracted from ultrasound-guided fine-needle aspiration biopsies of thyroid nodules were preserved in preservation solution and then submitted to a genetic testing company for *BRAF V600E* mutation testing. The *BRAF V600E* mutation test was performed by next-generation sequencing (NGS) at Ovison Gene Technology Tianjin Co., Ltd. using DNA-EZ Reagents F DNA-Be-Locked A for storage and transportation at room temperature.

### Construction and validation of the nomogram prediction model

A nomogram prediction model was constructed using a logistic regression algorithm, and a nomogram was plotted with the training data set. The goodness of fit between the observed and predicted values was examined using the Hosmer–Lemeshow method and is shown on the calibration curve plot [22]. The degree of identification and the consistency of the prediction model were examined by receiver operating characteristic (ROC) curves and calibration curves. The “rms” (ver. 6.3.0) and “pROC” (ver. 1.18.0) packages in R were applied to create the calibration curve and ROC graph [23]. The likelihood of CLNM was quantified as a risk score for predictive classification.

### 1D-CNN model building and training

The discrete variables (combined benign thyroid disease, nodule location, and capsular invasion) with multiple classifications were processed by the one-hot encoding method to make the distance between features more reasonable to calculate [24]. The numerical variables (age and maximum diameter of thyroid nodules) were z score normalized to make them comparable across features. In this study, 11 clinicopathological features (sex, age, combined benign thyroid disease, maximum diameter of thyroid nodules, nodule location, aspect ratio, microcalcification, nodule boundary, capsular invasion, *BRAF V600E* gene mutation and number of lesions) were incorporated in the construction of a one-dimensional convolutional neural network (1D-CNN) model, which was based on the PyTorch framework and realized by Python programming [25]. We constructed a 12-layer 1D-CNN, as shown in Supplementary Fig. 1, which includes one input layer, six 1D convolutional layers, one flattening layer, two dropout layers and two fully connected layers. The samples were divided into a training set and a test set at a ratio of 8:2. The training set was used to train the model, and the test set was used to validate the model. Eighty percent of the patients in each of the CLNM and nonmetastasis groups were randomly selected to form the training set, and the remaining 20% of the patients formed the validation set to ensure a balanced lymph node metastasis status in the training and validation sets. The 1D-CNN model was trained with cross-entropy as the loss function using the Adam optimizer. The learning rate was set to 0.0001, and the number of iterations was set to 200. After the model was trained, the test set samples were input into the model for prediction [26]. The area under ROC curve (AUC) was used to evaluate the predictive discriminability of the model [27].

### Subgroup analysis and validation

According to the preoperative ultrasound results, the 477 patients who participated in the model construction were divided into a subclinical metastasis group and a clinical metastasis group; those with lymph node metastasis indicated by preoperative ultrasound were included in the clinical metastasis group (*n* = 73), and those without lymph node metastasis were included in the subclinical metastasis group (*n* = 404). The constructed nomogram prediction model and CNN model were validated in these two groups (Table 2).

**Table 2:** Distribution of clinicopathological characteristics in the subclinical metastasis group and the clinical metastasis group

Characteristics	Overall N(%)	Subclinical metastasis group		Clinical metastasis group	
		Nonmetastasis N(%)	Metastasis N(%)	Nonmetastasis N(%)	Metastasis N(%)
Sample size	477	238	166	13	60
**Sex**					
Male	124 (26.0)	53 (22.27)	47 (28.31)	3 (23.08)	21 (35.00)
Female	353 (74.0)	185 (77.73)	119 (71.69)	10 (76.92)	39 (65.00)
**Age (years)**					
< 45	234 (49.1)	85 (35.71)	99 (59.64)	3 (23.08)	47 (78.33)
≥ 45	243(50.9)	153 (64.29)	67 (40.36)	10 (76.92)	13 (21.67)
**Combined benign thyroid disease**					
No	257 (53.9)	121 (50.84)	96 (57.83)	7 (53.85)	33 (55.00)
Nodular goiter	133 (27.9)	70 (29.41)	42 (25.30)	3 (23.08)	18 (30.00)
Lymphocytic thyroiditis	66 (13.8)	32 (13.45)	24 (14.46)	2 (15.38)	8 (13.33)
Both	21 (4.4)	15 (6.30)	4 (2.41)	1 (7.69)	1 (1.67)
**Surgical method**					
Unilateral	230 (48.2)	119 (50.00)	77 (46.39)	4 (30.77)	30 (50.00)
Total	247 (51.8)	119 (50.00)	89 (53.61)	9 (69.23)	30 (50.00)
***BRAF V600E* gene mutation**					
No	97 (20.3)	76 (31.93)	14 (8.43)	4 (30.77)	3 (5.00)
Yes	380 (79.7)	162 (68.07)	152 (91.57)	9 (69.23)	57 (95.00)
**Maximum diameter of thyroid nodules**					
< 10 mm	207 (43.4)	132 (55.46)	61 (36.75)	5 (38.46)	9 (15.00)
10–20 mm	201 (42.1)	86 (36.13)	79 (47.59)	8 (61.54)	28 (46.67)
> 20 mm	69 (14.5)	20 (8.40)	26 (15.66)	0 (0.00)	23 (38.33)
**Nodule location**					
Upper 1/3	98 (20.6)	49 (20.59)	30 (18.07)	4 (30.77)	15 (25.00)
Middle 1/3	145 (30.4)	75 (31.51)	51 (30.72)	4 (30.77)	15 (25.00)
Lower 1/3	76 (15.9)	34 (14.29)	32 (19.28)	2 (15.38)	8 (13.33)
Isthmus	62 (13.0)	33 (13.87)	23 (13.86)	2 (15.38)	4 (6.67)
Whole	96 (20.1)	47 (19.75)	30 (18.07)	1 (7.69)	18 (30.00)
**Aspect Ratio**					
< 1	274 (57.4)	132 (55.46)	98 (59.04)	7 (53.85)	37 (61.67)
≥ 1	203 (42.6)	106 (44.54)	68 (40.96)	6 (46.15)	23 (38.33)
**Microcalcification**					
No	123 (25.8)	70 (29.41)	40 (24.10)	2 (15.38)	11 (18.33)
Yes	354 (74.2)	168 (70.59)	126 (75.90)	11 (84.62)	49 (81.67)
**Nodule boundary**					
Clear	192 (40.3)	103 (43.28)	63 (37.95)	5 (38.46)	21 (35.00)
Blurred	285 (59.7)	135 (56.72)	103 (62.05)	8 (61.54)	39 (65.00)
**Capsular invasion**					
No	268 (56.2)	143 (60.08)	92 (55.42)	10 (76.92)	23 (38.33)
Close	63 (13.2)	39 (16.39)	20 (12.05)	0 (0.00)	4 (6.67)
Invasion	146 (30.6)	56 (23.53)	54 (32.53)	3 (23.08)	33 (55.00)
**Number of lesions**					
Unifocal	339 (71.1)	177 (74.37)	116 (69.88)	9 (69.23)	37 (61.67)
Multifocal	138 (28.9)	61 (25.63)	50 (30.12)	4 (30.77)	23 (38.33)
**ACR-TIRADS classification**					
3	19 (4.00)	7 (2.94)	9 (5.42)	0 (0.00)	3 (5.00)
4a	268 (56.2)	136 (57.14)	92 (55.42)	11 (84.62)	29 (48.33)
4b	149 (31.2)	70 (29.41)	55 (33.13)	2 (15.38)	22 (36.67)
4c	27 (5.66)	18 (7.56)	5 (3.01)	0 (0.00)	4 (6.67)
5	12 (2.52)	6 (2.52)	5 (3.01)	0 (0.00)	1 (1.67)
6	2 (0.42)	1 (0.42)	0 (0.00)	0 (0.00)	1 (1.67)

### Statistical analysis

Binary count data, unordered multicategorical count data, and ordered multicategorical count data were analyzed using the χ2 test. Binary logistic regression analysis was used to identify independent risk factors for CLNM. Then, a nomogram prediction model was constructed based on statistically significant indicators from the binary logistic regression analysis. Statistical analyses and the creation of the nomogram prediction model were performed with SPSS statistical software v26.0 and R Studio's R version 4.1.2 (R Project for Statistical Computing) [28], and 1D-CNN creation was performed with the machine learning software PyCharm community edition, based on Python language [25]. *P* < 0.05 indicates that the differences are statistically significant.

## Results

### CLNM is associated with several clinicopathological features

**Fig. 2:**
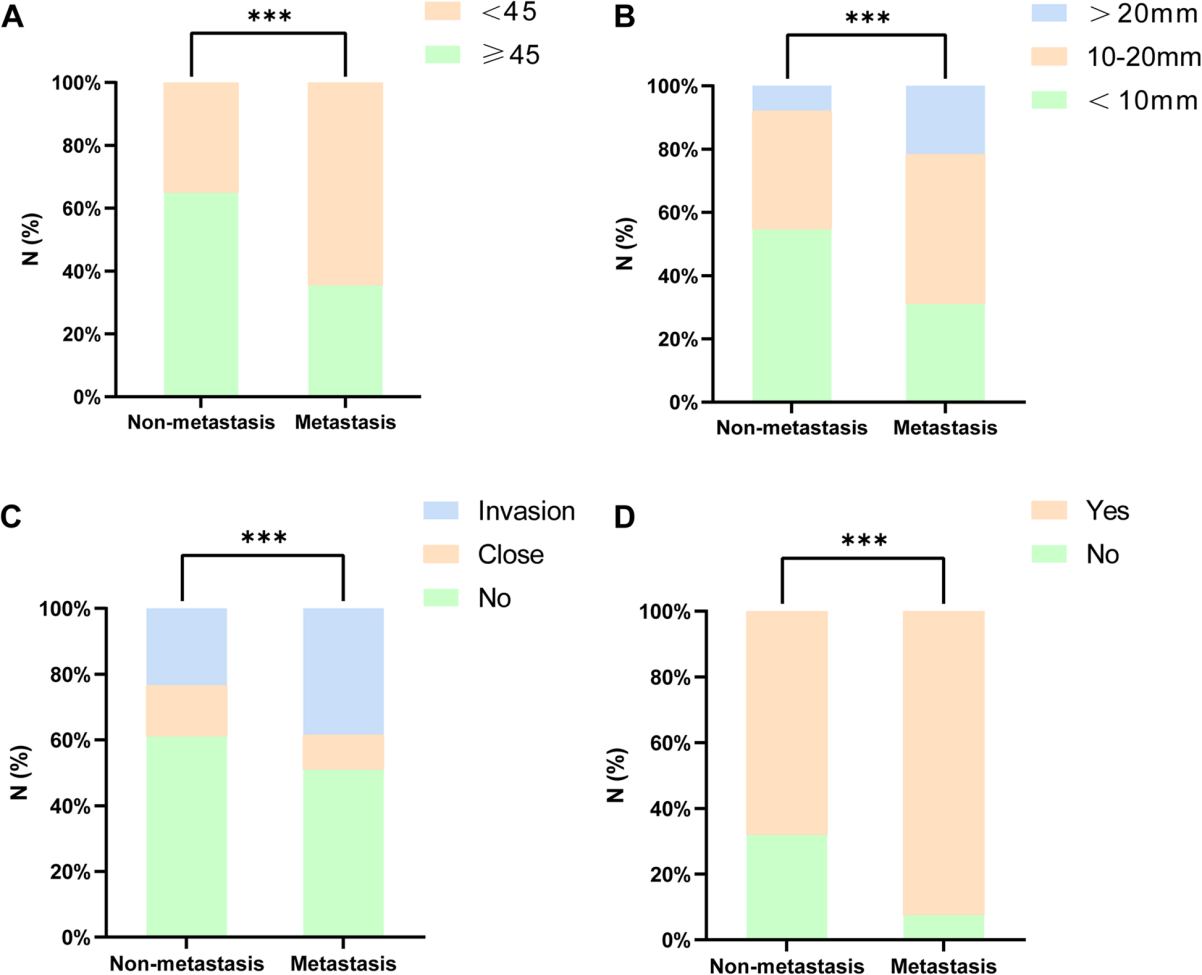
Correlation of central lymph node metastasis with age (2A), maximum diameter of thyroid nodules (2B), capsular invasion (2C), and *BRAF V600E* gene mutation (2D). The vertical axis indicates the proportion of the number of patients. ***stands for P ≤ 0.001

Among the 488 patients enrolled, 389 patients had the *BRAF V600E* gene mutation according to the FNAT-DNA *BRAF V600E* gene mutation results, with a gene mutation rate of 79.7% (Table 1). Eleven patients with LLNM only were excluded, and the remaining 477 patients were divided into groups with (*n* = 226) and without lymph node metastasis in the central region (*n* = 251) (Fig. 1). Fifty-three patients in the metastasis group also had LLNM. Univariate analysis revealed that the differences in age, maximum diameter of thyroid nodules, capsular invasion, and *BRAF V600E* mutation were statistically significant between the two groups (Fig. 2, *P* < 0.05). In contrast, the differences in sex, combined benign thyroid disease, surgical method, nodule location, aspect ratio, microcalcification, nodule boundary, number of lesions and ACR-TIRADS classification were not statistically significant between the two groups (Table 3, *P* > 0.05). Thus, CLNM was associated with age, maximum diameter of thyroid nodules, capsular invasion, and *BRAF V600E* gene mutation. In addition, multifactorial analysis further showed that age (OR = 3.380, *P* < 0.01), maximum diameter of thyroid nodules (OR = 2.228, *P* < 0.01; OR = 4.795, *P* < 0.01), *BRAF V600E* gene mutation (OR = 6.410, *P* < 0.01), and capsular invasion (OR = 1.507, *P* = 0.027) were independent risk factors for CLNM (Table 4).

**Table 3:** Correlation of central lymph node metastasis and clinicopathological features

Characteristics	Overall N(%)	Nonmetastasis N(%)	Metastasis N(%)	X^2^	*P -value*
Sample size	477	251	226		
**Sex**					
Male	124 (26.0)	56 (22.3)	68 (30.0)	3.739	0.053
Female	353 (74.0)	195 (77.7)	158 (70.0)		
**Age (years)**					
< 45	234 (49.1)	88 (35.1)	146 (64.6)	41.530	< 0.001
≥ 45	243(50.9)	163 (64.9)	80 (35.4)		
**Combined benign thyroid disease**					
No	257 (53.9)	128 (51.0)	129 (57.1)	5.803	0.122
Nodular goiter	133 (27.9)	73 (29.1)	60 (26.5)		
Lymphocytic thyroiditis	66 (13.8)	34 (13.5)	32 (14.2)		
Both	21 (4.4)	16 (6.4)	5 (2.2)		
**Surgical method**					
Unilateral	230 (48.2)	123 (49.0)	107 (47.3)	0.073	0.787
Total	247 (51.8)	128 (51.0)	119 (52.7)		
***BRAF V600E* gene mutation**					
No	97 (20.3)	80 (31.9)	17 (7.5)	43.527	< 0.001
Yes	380 (79.7)	171 (68.1)	209 (92.5)		
**Maximum diameter of thyroid nodules**					
< 10 mm	207 (43.4)	137 (54.6)	70 (31.0)	33.497	< 0.001
10–20 mm	201 (42.1)	94 (37.4)	107 (47.3)		
> 20 mm	69 (14.5)	20(8.0)	49 (21.7)		
**Nodule location**					
Upper 1/3	98 (20.6)	53 (21.1)	45 (19.9)	1.756	0.781
Middle 1/3	145 (30.4)	79 (31.5)	66 (29.2)		
Lower 1/3	76 (15.9)	36 (14.3)	40 (17.7)		
Isthmus	62 (13.0)	35 (14.0)	27 (12.0)		
Whole	96 (20.1)	48 (19.1)	48 (21.2)		
**Aspect Ratio**					
< 1	274 (57.4)	139 (55.4)	135 (59.7)	0.923	0.337
≥ 1	203 (42.6)	112 (44.6)	91 (40.3)		
**Microcalcification**					
No	123 (25.8)	72 (28.7)	51 (22.6)	2.327	0.127
Yes	354 (74.2)	179 (71.3)	175 (77.4)		
**Nodule boundary**					
Clear	192 (40.3)	108 (43.0)	84 (37.2)	1.698	0.193
Blurred	285 (59.7)	143 (57.0)	142 (62.8)		
**Capsular invasion**					
No	268 (56.2)	153 (61.0)	115 (50.9)	13.055	0.001
Close	63 (13.2)	39 (15.5)	24 (10.6)		
Invasion	146 (30.6)	59 (23.5)	87 (38.5)		
**Number of lesions**					
Unifocal	339 (71.1)	186 (74.1)	153 (67.7)	2.372	0.123
Multifocal	138 (28.9)	65 (25.9)	73 (32.3)		
**ACR-TIRADS classification**					
3	19 (4.00)	7 (2.79)	12 (5.31)	5.711	0.335
4a	268 (56.2)	147 (58.57)	121 (53.54)		
4b	149 (31.2)	72 (28.69)	77 (34.07)		
4c	27 (5.66)	18 (7.17)	9 (3.98)		
5	12 (2.52)	6 (2.39)	6 (2.66)		
6	2 (0.42)	1 (0.39)	1 (0.44)

**Table 4:** Multivariate logistic regression of factors associated with central lymph node metastasis

Multivariable risk factor for CLNM	β	SE	Wald	df	OR (95% CI)	P value
Age (< 45)	1.218	0.192	40.260	1	3.380 (0.181–0.417)	< 0.01
Maximum diameter of thyroid nodules (< 10 mm)			31.853	2		
10–20 mm	0.801	0.204	15.435	1	2.228 (0.094–0.367)	< 0.01
> 20 mm	1.568	0.303	26.712	1	4.795 (0.199–0.761)	< 0.01
Capsular invasion (no)	0.410	0.186	4.882	1	1.507 (1.314–1.864)	0.027
*BRAF V600E* gene mutation (no)	1.858	0.312	35.505	1	6.410 (3.757–13.245)	< 0.01

*Β* regression coefficient, *SE* standard error, *df* degrees of freedom, *OR* odds ratio. Factor items in parentheses are controls

### Construction of the nomogram prediction model

**Fig. 3:**
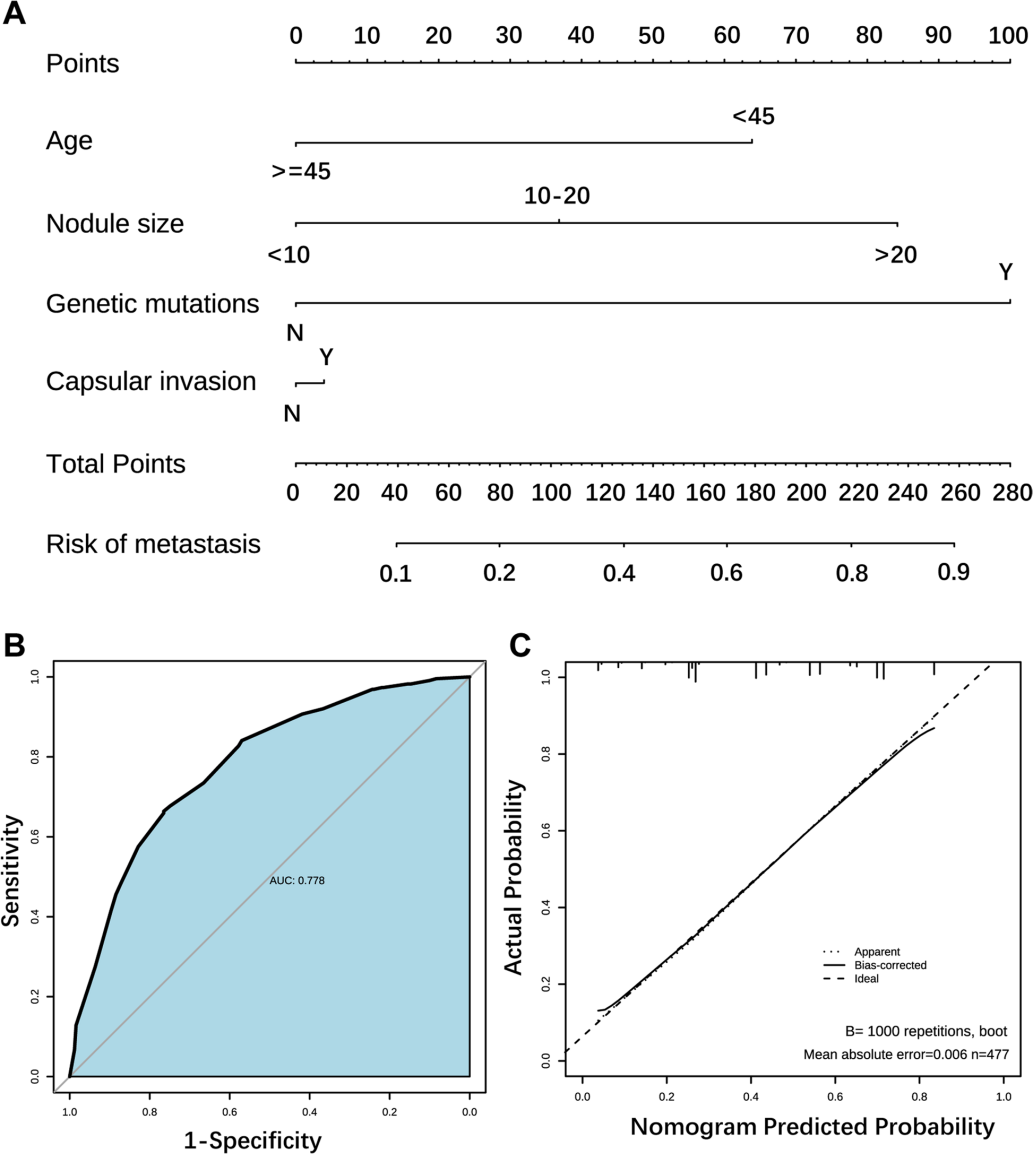
Construction of the nomogram prediction model. 3**A**: Nomogram of the logistic regression model for predicting central lymph node metastasis; 3**B**: ROC curve plot; 3**C**: Prediction model calibration curve

**Fig. 4:**
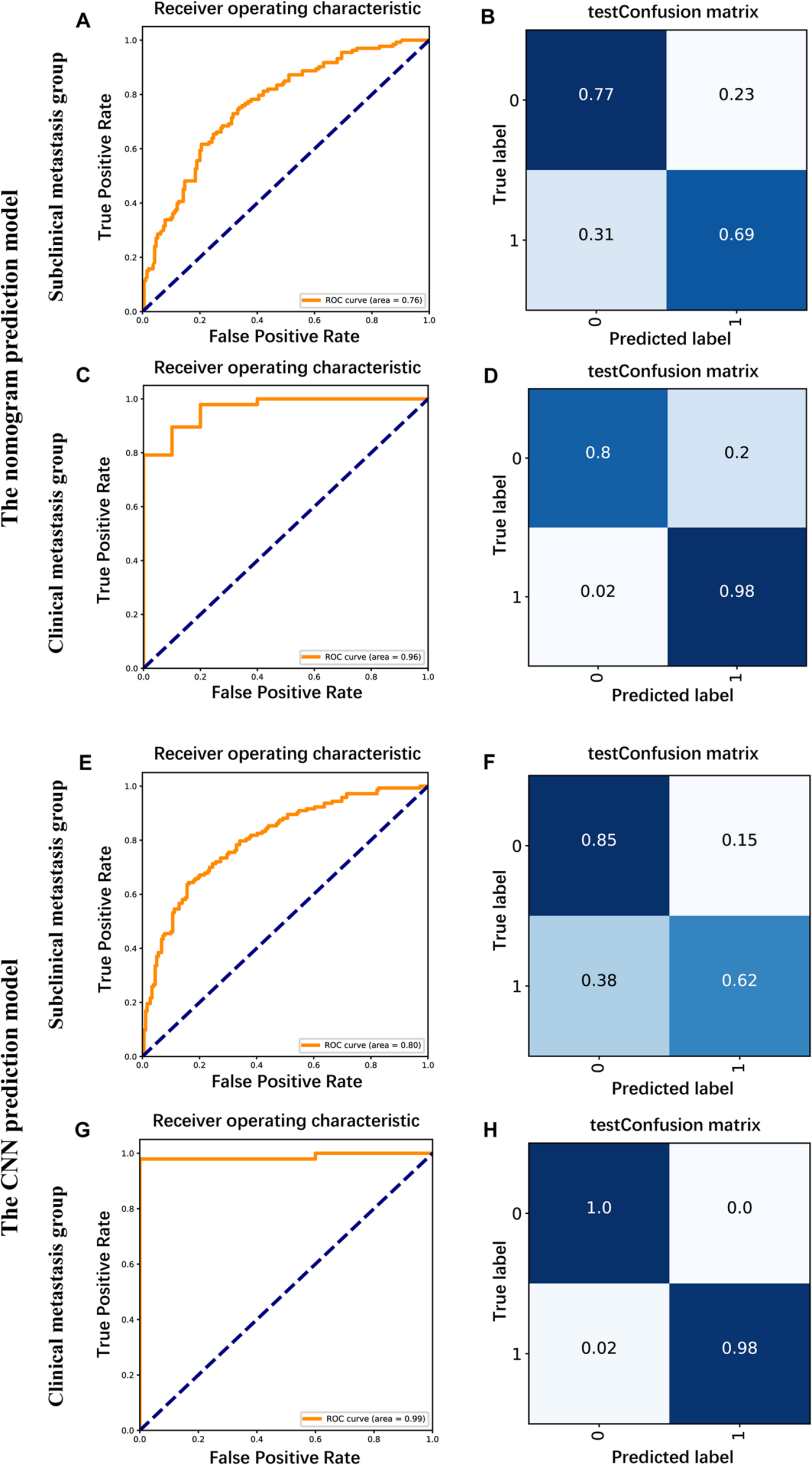
Subgroup analysis and validation. 4**A**-**B**: ROC curves (**A**) and diagnostic efficacy evaluation indexes (**B**) of the nomogram prediction model in subclinical metastasis group; 4**C**-**D**: ROC curves (**C**) and diagnostic efficacy evaluation indexes (**D**) of the nomogram prediction model in clinical metastasis group; 4**E**-**F**: ROC curves (**E**) and diagnostic efficacy evaluation indexes (**F**) of the CNN prediction model in subclinical metastasis group; 4G-H: ROC curves (**G**) and diagnostic efficacy evaluation indexes (**H**) of the CNN prediction model in clinical metastasis group

The statistically significant indicators (age, maximum diameter of thyroid nodules, capsular invasion and *BRAF V600E* mutation) from the multifactorial analysis were used to construct a nomogram prediction model for CLNM. The sum of the points for each feature equals the probability of CLNM of PTC (Fig. 3A). To characterize the predictive efficacy of the model, we performed ROC curve analysis, and the AUC value of the model was 0.778 (95% CI: 0.7374–0.8196; *P* < 0.001). The calibration curve of the model showed good agreement between the predicted and observed results (Fig. 3B-C). In addition, we divided the enrolled patients into two subgroups for validation (Table 2), and the AUC value of the model in the subclinical metastasis group was 0.76, and that in the clinical metastasis group was 0.96, both indicating high diagnostic efficacy (Fig. 4A-D).

### Construction of the CNN prediction model

**Fig. 5:**
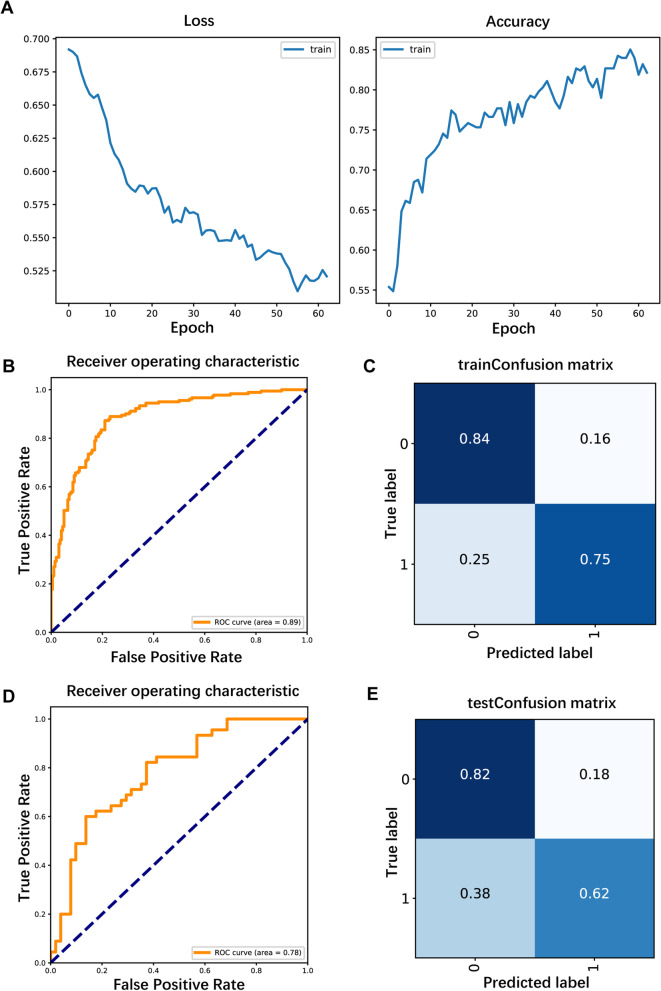
Construction of the convolutional neural network prediction model. 5A: Iterative plot of the convolutional neural network prediction model; 5B, 4D: ROC curves obtained with the training and test sets; 5C, 5E: Sensitivity, specificity, false positive rate and false negative rate obtained with the training and test sets

Next, we incorporated all the indicators to build a CNN prediction model for CLNM (Table 5). The iterative graph of this model showed that the loss of the training model decreased as the number of iterations increased. Figure 5A indicates that the accuracy of the training model increased as the number of iterations increased, and the prediction model reached the minimum loss of training data and the maximum accuracy after 60 iterations of training. ROC curve analysis in the training set showed that the model had high predictive efficacy; the AUC value was as high as 0.89, the specificity and sensitivity were 0.84 and 0.75, and the false positive and false negative rates were 0.16 and 0.25 (Fig. 5B-C). In the test set, the AUC value was 0.78, the specificity and sensitivity were 0.82 and 0.62, and the false positive and false negative rates were 0.18 and 0.38, suggesting that the model has good predictive efficacy (Fig. 5D-E). We also validated the model in the subclinical metastasis group and the clinical metastasis group (Table 2), and the AUC value of the model were 0.8 in the subclinical metastasis group and 0.99 in the clinical metastasis group, suggesting that the model has high diagnostic efficacy. Notably, the model had a high specificity (85%) in the subclinical metastasis group and a high sensitivity (98%) in the clinical metastasis group (Fig. 4E-H).

**Table 5:** Convolutional neural network baseline table

	Training set		Test set		X^2^	*P -value*
	N	%	N	%		
**Sex**						
Male	92	24.1%	32	33.7%	3.163	0.075
Female	290	75.9%	63	66.3%		
**Age (years)**						
< 45	184	48.2%	50	52.6%	0.441	0.507
≥ 45	198	51.8%	45	47.4%		
**Combined benign thyroid disease**						
No	209	54.7%	48	50.5%	2.126	0.547
Nodular goiter	101	26.4%	32	33.7%		
Lymphocytic thyroiditis	55	14.4%	11	11.6%		
Both	17	4.5%	4	4.2%		
***BRAF V600E* gene mutation**						
No	79	20.7%	18	18.9%	0.054	0.816
Yes	303	79.3%	77	81.1%		
**Lymph node metastasis**						
No	201	52.6%	50	52.6%	< 0.001	1.000
Yes	181	47.4%	45	47.4%		
**Maximum diameter of thyroid nodules**						
< 10 mm	167	43.7%	40	42.1%	0.542	0.763
10–20 mm	162	42.4	39	41.1%		
> 20 mm	53	13.9%	16	16.8%		
**Nodule location**						
Upper 1/3	79	20.7%	19	20.0%	1.573	0.814
Middle 1/3	118	30.9%	27	28.4%		
Lower 1/3	63	16.5%	13	13.7%		
Isthmus	49	12.8%	13	13.7%		
Whole	73	19.1%	23	24.2%		
**Aspect Ratio**						
< 1	230	60.2%	44	46.3%	5.453	0.020
≥ 1	152	39.8%	51	53.7%		
**Microcalcification**						
No	101	26.4%	22	23.2%	0.274	0.601
Yes	281	73.6%	73	76.8%		
**Nodule boundary**						
Clear	158	41.4%	34	35.8%	0.764	0.382
Blurred	224	58.6%	61	64.2%		
**Capsular invasion**						
No	218	57.1%	50	52.6%	5.020	0.081
Close	55	14.4%	8	8.4%		
Invasion	109	28.5%	37	38.9%		
**Number of lesions**						
Unifocal	276	72.3%	63	66.3%	1.031	0.310
Multifocal	106	27.7%	32	33.7%

### Stratified analysis according to nodule size

**Fig. 6:**
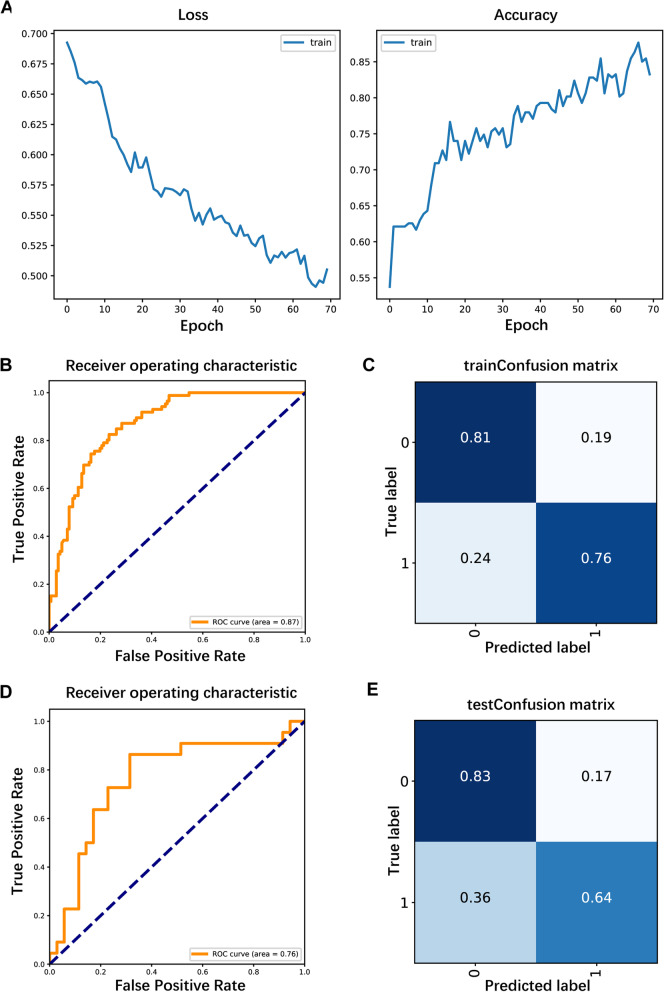
Construction of convolutional neural network prediction models for patients with nodule diameters ≤ 1 cm. 6**A**: Iterative plot of the convolutional neural network prediction model; 6**B**, **D**: ROC curves obtained with the training and test sets; 6**C**, **E**: Sensitivity, specificity, false positive rate and false negative rate obtained with the training and test sets

We next constructed another CNN model for the population (284 patients) with thyroid nodules measuring less than or equal to 1 cm in diameter to assess its predictive value for this specific subgroup. The prediction model also had high performance, and its accuracy in the training set increased as the number of iterations increased. After 70 iterations, the training data loss was minimized, and the accuracy was maximized (Fig. 6A). The AUC values were 0.87 and 0.76 for the training and test sets, with specificities of 0.81 and 0.83, sensitivities of 0.76 and 0.64, false positive rates of 0.19 and 0.17, and false negative rates of 0.24 and 0.36, respectively (Fig. 6B-E). Therefore, the model could be a good predictor of CLNM in patients with nodule diameters measuring ≤ 1 cm.

## Discussion

In this study, we retrospectively collected data from 488 patients with PTC and investigated the correlations between the CLNM and clinicopathological features using univariate analysis and binary logistic regression analysis. We constructed a nomogram prediction model and a CNN prediction model for CLNM, thus facilitating the formulation of individualized patient treatment plans and assisting clinicians in accurately diagnosing the disease.

PTC is usually characterized by chromosomal rearrangements of RET or point mutations in the RAS or BRAF proto-oncogene, and mutations in the BRAF, RAS, or RET genes are found in nearly 70% of PTC cases [19, 29]. Of these, BRAF mutations are seen in 60%-70% of PTCs, making them the most common mutations in PTC [30, 31]. Yan et al. showed that 1715 of 2048 patients with PTC had *BRAF V600E* mutations, with a mutation rate of 83.7% [32]. In the present study, the *BRAF V600E* gene mutation rate was as high as 79.71%. There is some controversy in previous studies regarding the correlation between the *BRAF V600E* gene mutation and cervical lymph node metastasis [33–39], and our study showed that the *BRAF V600E* gene mutation (OR = 6.410, *P* < 0.001) was an independent risk factor for CLNM, suggesting that it may serve as an important reference in predicting lymph node metastasis.

In further analyses, in addition to the *BRAF V600E* mutation, we also found that age, maximum diameter of thyroid nodules, and capsular invasion were also independent risk factors for CLNM. The risk of CLNM was higher in younger patients and those with larger diameter nodules and with capsular invasion. The prediction model developed by incorporating these independent risk factors into the logistic regression model had good predictive efficacy. The CNN is a typical, deep learning-based feedforward neural network that employs convolutional computations and a deep structure, has a good self-learning ability, adaptive performance and fault tolerance, and can automatically extract features from the input data and use them for further classification or prediction [40, 41]. Of the different kinds of CNNs, a 1D-CNN is mainly applied to the data processing of sequence classes [42–44]; therefore, the 1D-CNN framework was used to automatically identify all clinical variables and indicators of enrolled patients. Compared with the regression prediction model, the 1D-CNN model has a more accurate prediction capability and achieves a perfect interaction between AI algorithms and medical diagnosis. Here, it was used as the first application of deep learning in the prediction of CLNM of PTC.

A retrospective analysis of 500 thyroid nodules (maximum nodule diameter ≤ 2.0 cm, TI-RADS classification 4c) examined by ultrasound showed that an anteroposterior diameter of 0.9 cm could be used as a threshold for assessing the risk of metastasis of malignant thyroid nodules [45]. In this study, our results showed that the risk of CLNM was 2.228 times higher in patients with thyroid nodules 10–20 mm in maximum diameter than in patients with thyroid nodules < 10 mm in maximum diameter and 4.795 times higher in patients with thyroid nodules > 20 mm than in patients with thyroid nodules < 10 mm in maximum diameter. Therefore, the larger the maximum diameter of the thyroid nodule is, the higher the rate of CLNM. We usually only treat patients with thyroid nodule diameters < 10 mm through observation in clinical practice. Thus, treatment may be delayed for those patients who also have CLNM. Better means of lymph node detection are limited, so it is crucial to accurately predict lymph node metastasis in this group of patients. We developed a predictive model with high predictive efficacy for this population that can accurately distinguish between high- and low-risk clinical groups, assist in determining the follow-up treatment, and thus improve the prognosis of patients. The high specificity of the model is also useful for screening subsets of patients for clinical observation.

The subgroup analysis and validation of this study showed that the nomogram prediction model and the CNN prediction model had high specificity in the subclinical metastasis group, suggesting that our model in cN0 patients can better predict patients who eventually do not have CLNM, thus enabling these patients to avoid unnecessary PCND and reduce complications. In addition, our prediction models had high sensitivity in the clinical metastasis group, suggesting that the models can better predict patients who actually have metastasis in clinically lymph node-positive patients, thus providing a reference for clinical diagnosis.

Multifocal papillary thyroid carcinoma (MPTC) accounts for 20.0%-40.0% of PTCs [46]. Compared with unifocal PTC, MPTC is more malignant, more aggressive and has a higher rate of neck lymph node metastasis. Some studies have shown that the rate of CLNM in MPTC ranges from 23.3% to 58.5%, and there is a high risk of recurrence and poor prognosis after surgery [47]. Tam et al. studied the clinicopathological data of 912 patients with PTC and found that patients with multifocal PTC and total tumor diameters > 1 cm had a significantly higher risk of CLNM than patients with unifocal PTC [48], and some studies found multifocality to be an independent risk factor for CLNM in PTC [49, 50]. In this study, the number of lesions did not significantly differ between the CLNM group and the nonmetastasis group, probably because most of the enrolled patients were unifocal PTC patients and there were more patients with tumor diameters less than 1 cm, which may have produced some bias in the results. Thus, the correlation between multifocality and CLNM in PTC patients still needs to be confirmed in a large sample and multicenter clinical study.

Although we developed a new prediction model to diagnose CLNM in patients with PTC, there are still some limitations. The high rate of *BRAF V600E* gene mutation may be related to ethnicity or race, and further validation of the *BRAF V600E* gene mutation in tissues should be performed in surgical specimens. *BRAF V600E* is not solely responsible for PTC aggressiveness and further research on mutations coexisting with *BRAF V600E* is needed [51]. In addition, the prediction model of this study needs to be further validated in multicenter and large-sample studies.

In conclusion, we innovatively established a prediction model based on genetic mutations and clinicopathological factors that can be used to evaluate CLNM of PTC with high sensitivity and specificity, thus achieving the ideal combination of molecular and traditional diagnostic methods for clinical application.

## Supplementary Information

**Additional file 1.**

## Data Availability

The datasets used and analysed during the current study available from the corresponding author on reasonable request.

## Abbreviations

AI: Artificial intelligence
AUC: Area under the curve
CLNM: Central lymph node metastasis
cN0: Clinically lymph node-negative
CNN: Convolutional Neural Network
DFS: Disease-free survival
FNAT-DNA: Fine-needle aspiration tissue DNA
LLND: Lateral lymph node dissection
LLNM: Lateral neck lymph node metastasis
MPTC: Multifocal papillary thyroid carcinoma
NGS: Next-generation sequencing
1D-CNN: One-dimensional convolutional neural network
OS: Overall survival
PCND: Prophylactic central neck dissection
PTC: Papillary thyroid carcinoma
ROC: Receiver operating characteristic

## References

[1] LiVolsi VA (2011). Papillary thyroid carcinoma: an update. Mod Pathol.

[2] Filetti S (2019). Thyroid cancer: ESMO Clinical Practice Guidelines for diagnosis, treatment and follow-up†. Ann Oncol.

[3] Shi L (2021). The effect of the area proportion of the metastatic lesion within the central metastatic lymph node on response to therapy in papillary thyroid carcinoma. Oncol Lett.

[4] Zhao H, Huang T, Li H (2019). Risk factors for skip metastasis and lateral lymph node metastasis of papillary thyroid cancer. Surgery.

[5] Seok J (2021). Factors Affecting Central Node Metastasis and Metastatic Lymph Node Ratio in Papillary Thyroid Cancer. Otolaryngol Head Neck Surg.

[6] Zhao H, Li H (2019). Meta-analysis of ultrasound for cervical lymph nodes in papillary thyroid cancer: Diagnosis of central and lateral compartment nodal metastases. Eur J Radiol.

[7] Xing Z (2020). Thyroid cancer neck lymph nodes metastasis: Meta-analysis of US and CT diagnosis. Eur J Radiol..

[8] Ji YB (2014). Accuracy of intraoperative determination of central node metastasis by the surgeon in papillary thyroid carcinoma. Otolaryngol Head Neck Surg.

[9] Mulla M, Schulte K-M (2012). Central cervical lymph node metastases in papillary thyroid cancer: a systematic review of imaging-guided and prophylactic removal of the central compartment. Clin Endocrinol (Oxf).

[10] Arora N (2009). Papillary thyroid carcinoma and microcarcinoma: is there a need to distinguish the two?. Thyroid.

[11] Su H, Li Y (2019). Prophylactic central neck dissection and local recurrence in papillary thyroid microcarcinoma: a meta-analysis. Braz J Otorhinolaryngol.

[12] Giordano D (2012). Complications of central neck dissection in patients with papillary thyroid carcinoma: results of a study on 1087 patients and review of the literature. Thyroid.

[13] Alsubaie KM (2021). Prophylactic Central Neck Dissection for Clinically Node-Negative Papillary Thyroid Carcinoma. Laryngoscope.

[14] Scherl S (2014). The effect of surgeon experience on the detection of metastatic lymph nodes in the central compartment and the pathologic features of clinically unapparent metastatic lymph nodes: what are we missing when we don't perform a prophylactic dissection of central compartment lymph nodes in papillary thyroid cancer?. Thyroid.

[15] Carmel-Neiderman NN (2021). Prophylactic central neck dissection has no advantage in patients with metastatic papillary thyroid cancer to the lateral neck. J Surg Oncol.

[16] Gambardella C (2016). Clinical significance of prophylactic central compartment neck dissection in the treatment of clinically node-negative papillary thyroid cancer patients. World J Surg Oncol.

[17] Forman, A. & Sotelo, J. Tumor-Based Genetic Testing and Familial Cancer Risk. Cold Spring Harb Perspect Med. 2020;10. 10.1101/cshperspect.a036590. 10.1101/cshperspect.a036590 PMC7397843 31570381

[18] Lan X (2020). Genomic landscape of metastatic papillary thyroid carcinoma and novel biomarkers for predicting distant metastasis. Cancer Sci.

[19] Xing M, Haugen BR, Schlumberger M (2013). Progress in molecular-based management of differentiated thyroid cancer. Lancet.

[20] Li R (2021). Correlation between US-FNAC with BRAF V600E Mutation Analysis and Central Neck Lymph Node Metastasis in cN0 Papillary Thyroid Cancer. Biomed Res Int.

[21] Song J-Y (2018). Predictive Value of BRAF Mutation for Lymph Node Metastasis in Papillary Thyroid Cancer: A Meta-analysis. Curr Med Sci.

[22] Paul P, Pennell ML, Lemeshow S (2013). Standardizing the power of the Hosmer-Lemeshow goodness of fit test in large data sets. Stat Med.

[23] Robin X (2011). pROC: an open-source package for R and S+ to analyze and compare ROC curves. BMC Bioinformatics.

[24] Okada S, Ohzeki M, Taguchi S (2019). Efficient partition of integer optimization problems with one-hot encoding. Sci Rep.

[25] Perkel J (2015). M. Programming: Pick up Python. Nature.

[26] Rala Cordeiro, J., Raimundo, A., Postolache, O. & Sebastião, P. Neural Architecture Search for 1D CNNs-Different Approaches Tests and Measurements. Sensors (Basel). 2021;21. 10.3390/s21237990. 10.3390/s21237990 PMC8659883 34883994

[27] Mandrekar JN (2010). Receiver operating characteristic curve in diagnostic test assessment. J Thorac Oncol.

[28] Dessau RB, Pipper CB (2008). "R"–project for statistical computing. Ugeskr Laeger.

[29] Kimura ET (2003). High prevalence of BRAF mutations in thyroid cancer: genetic evidence for constitutive activation of the RET/PTC-RAS-BRAF signaling pathway in papillary thyroid carcinoma. Cancer Res.

[30] Haddad RI (2018). NCCN Guidelines Insights: Thyroid Carcinoma, Version 2.2018. J Natl Compr Canc Netw.

[31] Cho, Y. Y. et al. Highly Sensitive and Specific Molecular Test for Mutations in the Diagnosis of Thyroid Nodules: A Prospective Study of -Prevalent Population. Int J Mol Sci 21, doi:10.3390/ijms21165629 (2020). 10.3390/ijms21165629 PMC7460614 32781560

[32] Yan C, Huang M, Li X, Wang T, Ling R (2019). Relationship between BRAF V600E and clinical features in papillary thyroid carcinoma. Endocr Connect.

[33] Zhao L (2016). Concomitant high expression of BRAFV600E, P-cadherin and cadherin 6 is associated with High TNM stage and lymph node metastasis in conventional papillary thyroid carcinoma. Clin Endocrinol (Oxf).

[34] Guo L (2019). Role of ultrasonographic features and quantified BRAFV600E mutation in lymph node metastasis in Chinese patients with papillary thyroid carcinoma. Sci Rep.

[35] Kurtulmus N (2016). BRAF Mutation: Has It a Role in Cervical Lymph Node Metastasis of Papillary Thyroid Cancer?. Eur Thyroid J.

[36] Zhou C, Li J, Wang Y, Xue S, Zhang Y (2019). Association of BRAF gene and TSHR with cervical lymph node metastasis of papillary thyroid microcarcinoma. Oncol Lett.

[37] Zhan J (2020). Prediction of cervical lymph node metastasis with contrast-enhanced ultrasound and association between presence of BRAF and extrathyroidal extension in papillary thyroid carcinoma. Ther Adv Med Oncol.

[38] Kim S-J (2012). BRAF V600E mutation is associated with tumor aggressiveness in papillary thyroid cancer. World J Surg.

[39] Nam JK (2012). Is the BRAF(V600E) mutation useful as a predictor of preoperative risk in papillary thyroid cancer?. Am J Surg.

[40] Li, Z., Liu, F., Yang, W., Peng, S. & Zhou, J. A Survey of Convolutional Neural Networks: Analysis, Applications, and Prospects. IEEE Trans Neural Netw Learn Syst PP. 2021. 10.1109/TNNLS.2021.3084827. 10.1109/TNNLS.2021.3084827 34111009

[41] Alzubaidi L (2021). Review of deep learning: concepts, CNN architectures, challenges, applications, future directions. J Big Data.

[42] Ma D (2021). Classifying breast cancer tissue by Raman spectroscopy with one-dimensional convolutional neural network. Spectrochim Acta A Mol Biomol Spectrosc.

[43] Hsieh CH, Li YS, Hwang BJ. & Hsiao CH. Detection of Atrial Fibrillation Using 1D Convolutional Neural Network. Sensors (Basel). 2020;20. 10.3390/s20072136. 10.3390/s20072136 PMC7180882 32290113

[44] Kopylov AT (2021). Convolutional neural network in proteomics and metabolomics for determination of comorbidity between cancer and schizophrenia. J Biomed Inform.

[45] Huang K (2018). The anteroposterior diameter of nodules in the risk assessment of papillary thyroid microcarcinoma. Medicine (Baltimore).

[46] So YK, Kim MW, Son Y-I (2015). Multifocality and bilaterality of papillary thyroid microcarcinoma. Clin Exp Otorhinolaryngol.

[47] Yin X (2017). Influence of tumor extent on central lymph node metastasis in solitary papillary thyroid microcarcinomas: a retrospective study of 1092 patients. World J Surg Oncol.

[48] Tam AA (2016). Association of multifocality, tumor number, and total tumor diameter with clinicopathological features in papillary thyroid cancer. Endocrine.

[49] Kim HJ, Sohn SY, Jang HW, Kim SW, Chung JH (2013). Multifocality, but not bilaterality, is a predictor of disease recurrence/persistence of papillary thyroid carcinoma. World J Surg.

[50] Feng J-W (2020). Significance of multifocality in papillary thyroid carcinoma. Eur J Surg Oncol.

[51] Zhao L (2020). The coexistence of genetic mutations in thyroid carcinoma predicts histopathological factors associated with a poor prognosis: a systematic review and network meta-analysis. Front Oncol..

