# Supplementary figures and images for: Deep learning-based multifeature integration robustly predicts central lymph node metastasis in papillary thyroid cancer
Source: BMC Cancer. 2023 Feb 8;23:128. doi: 10.1186/s12885-023-10598-8 (PMC9906958; doi:10.1186/s12885-023-10598-8)

**Supplementary Figure 1:** Structure of the one-dimensional convolution neural network model.


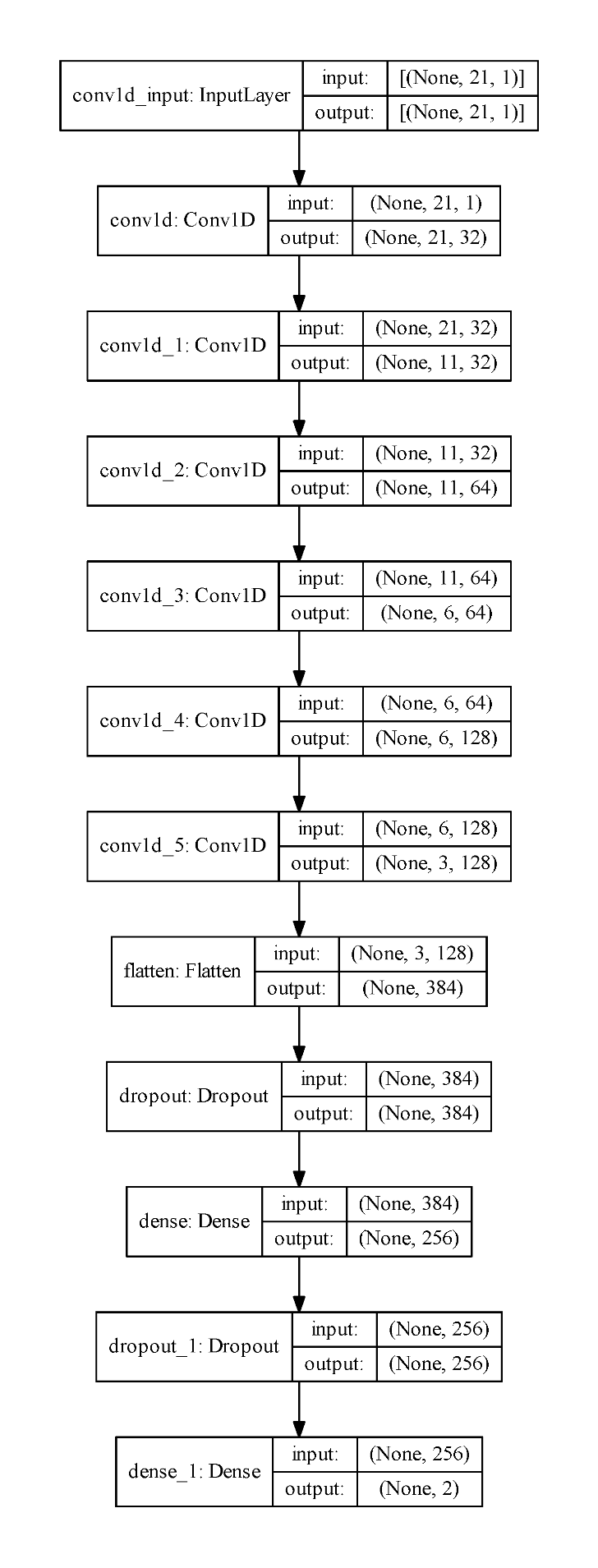

Supplement: Supplementary file 1 — Additional file 1. [file 12885_2023_10598_MOESM1_ESM.docx]
